# Relationship Between Brazilian Dietary Patterns and the Global Syndemic: Data from the CUME Study

**DOI:** 10.3390/ijerph22050805

**Published:** 2025-05-21

**Authors:** Jéssica Bevenuto Mattar, Marcos Heil Costa, Ana Luiza Gomes Domingos, Helen Hermana Miranda Hermsdorff, Adriano Marçal Pimenta, Josefina Bressan

**Affiliations:** 1 Department of Nutrition and Health, Universidade Federal de Viçosa (UFV), Viçosa 36570-900, MG, Brazil; helenhermana@ufv.br (H.H.M.H.); jbrm@ufv.br (J.B.); 2 Department of Agricultural Engineering, Universidade Federal de Viçosa (UFV), Viçosa 36570-900, MG, Brazil; mhcosta@ufv.br; 3 School of Arts, Sciences and Humanities, University of São Paulo, 1000 Arlindo Bettio Avenue, São Paulo 03828-000, SP, Brazil; algdomingos@gmail.com; 4 Department of Nursing, Universidade Federal do Paraná (UFPR), Curitiba 80035-050, PR, Brazil; adriano.pimenta@ufpr.br

**Keywords:** obesity, undernutrition, carbon footprint, water footprint, ecological footprint, sustainable diet

## Abstract

Global food systems are contributing to a shift toward unhealthy diets, which is linked to the three components of the global syndemic. This cross-sectional study evaluates how dietary patterns in Brazil are associated with the components of the global syndemic. Anthropometric and food intake data were obtained from the CUME Study—a prospective cohort conducted with a sample of Brazilian university graduates. BMI was used to assess obesity. Insufficient intake of micronutrients was considered undernutrition. Carbon, water, and ecological footprints were used to assess the environmental impact of dietary patterns. Dietary patterns were identified through principal components analysis. Linear regression models were used to evaluate associations between dietary patterns and the components of the global syndemic. The Unhealthy Dietary Pattern was positively associated with BMI and had the highest environmental impact. The Brazilian Dietary Pattern was also positively associated with BMI but had the lowest environmental impact. The Healthy Dietary Pattern was the most protective against micronutrient inadequacy. Diet affected the environment and people’s health in this sample. The dietary patterns identified here as contributing to poor health and environmental damage can help the government develop policies that incorporate the costs of these effects into the prices of food.

## 1. Introduction

A syndemic is defined as two or more diseases that co-occur in time and place; interact with each other at biological, psychological, or social levels; and share common underlying determinants. In this sense, the global syndemic is a concept that defines the interconnection between obesity, undernutrition, and climate change, the three greatest threats to human and planetary health [1].

While food demand worldwide is increasing, and global food systems can produce enough food to feed the global population, hunger and food insecurity persist while obesity rates are rising [2,3,4,5]. This is because current global food systems are shifting toward a less diverse, low-quality diet rich in animal-source and ultra-processed foods [1,6,7]. In Brazil, a country of continental dimensions with remarkable biodiversity, the dietary pattern has been undergoing a process of standardization in which ten products account for more than 45% of food consumption: rice, beans, white bread, beef, chicken, banana, milk, soft drinks, beers, and sugar [8].

Additionally, food systems contribute to about a third of global greenhouse gas emissions that cause climate change because of land use/land-use change activities (71%), with the remainder coming from supply chain activities: retail, transport, consumption, fuel production, waste management, industrial processes, and packaging [9]. Consequently, food systems are one of the underlying factors contributing to the global syndemic’s three components.

Changes in global dietary patterns adversely affect human and planetary health [10]. Recent efforts to evaluate the sustainability of diets globally have yielded more nuanced metrics for their environmental impacts [4,11]. Studies on the individual impact of food, food groups, and dietary patterns on health [12,13,14] and the environment [11,15,16,17] have been conducted, but none have focused on the assessment of dietary patterns on the three components of the global syndemic at once. Therefore, this study aimed to evaluate how dietary patterns in Brazil are associated with the components of the global syndemic.

## 2. Materials and Methods

### 2.1. CUME Study

This study used baseline data from the Cohort of Universities of Minas Gerais (CUME Study) carried out biennially since 2016 [18]. Alumni of seven Federal Universities from Minas Gerais State in Brazil were invited to participate in the study. Once registered, participants had access to the baseline questionnaire divided into two phases. The first phase included questions related to lifestyle; sociodemographic, anthropometric, biomedical, and clinical data; individual and family morbidity; and personal examination history.

In the second phase, participants completed a semi-quantitative Food Frequency Questionnaire (FFQ) validated for the study population [19]. The FFQ included 144 food items grouped into dairy, meat and fish, cereals and leguminous, fats and oils, fruits and vegetables, beverages, and other foods. Participants reported each item’s frequency, number of times consumed, and portion size over the previous 12 months.

### 2.2. Participants

Between 2016 and 2022, 8694 participants completed the baseline questionnaire. Individuals who resided abroad in the year before completing the questionnaire (*n* = 338), non-Brazilians (*n* = 54), individuals over 60 years old (*n* = 187), women who reported being pregnant in the year before completing the questionnaire (*n* = 308), those who did not answer questions about their weight or height (*n* = 12), and individuals who consumed either <500 or ≥6000 kcal/day (*n* = 235) [20,21] were not included in the analysis. Thus, the final sample of this study is composed of 7560 individuals.

### 2.3. Malnutrition Outcome Variables

#### 2.3.1. Obesity

Data on weight (kg) and height (m) were self-reported in the first phase of the questionnaire and used to calculate body mass index (BMI) (kg/m^2^) [22]. Obesity was defined as a BMI ≥ 30 kg/m^2^ [23].

#### 2.3.2. Undernutrition

Insufficient intake of micronutrients was defined as undernutrition. The FFQ data were processed, and the amount of each food item consumed was measured in grams or milliliters per day. Nutrient intake was then determined using Brazilian nutritional composition tables as a reference [24,25]. Mean daily intakes of vitamin A; thiamin (B1); riboflavin (B2); niacin (B3); pyridoxine (B6); folate (B9); cobalamin (B12); C, D, and E, as well as the minerals iron (Fe), magnesium (Mg), zinc (Zn), selenium (Se), copper (Cu), calcium (Ca), and potassium (K), obtained through FFQ data, were compared and classified as adequate or inadequate, according to the Dietary Reference Intake (DRI) values of the Institute of Medicine. The Estimated Average Requirement (EAR) was used for all nutrients investigated here, except for K, which was evaluated as Adequate Intake (AI) [26,27,28,29,30,31].

Then, the total inadequacy of all vitamins was calculated to create a score for vitamin inadequacy, the total inadequacy of all minerals was calculated to create a score for mineral inadequacy, and the total inadequacy of all micronutrients (vitamins and minerals) was calculated to create a general score for micronutrient inadequacy.

### 2.4. Environmental Outcome Variables

The carbon (CFP), water (WFP), and ecological (EFP) footprints of each individual were calculated by multiplying the average amount of each food by footprint coefficients [32] that quantify, respectively, the emissions of greenhouse gases (expressed in grams of carbon dioxide equivalent (gCO2e)), the use of total water (including the blue, green, and gray water, expressed as volume (L)), and the demand from the regenerative capacity of the biosphere (expressed in units of land area (m^2^)). All environmental footprints were calculated per gram or milliliter of food, per person, per day.

### 2.5. Exposure Assessment

#### Dietary Patterns

In this study, to identify the major dietary patterns and reduce the complexity of the data, the FFQ food items were first grouped into 27 groups, considering their nutritional composition and the level and intent of processing, according to the NOVA classification (unprocessed and minimally processed foods; processed culinary or food industry ingredients; processed food; and ultra-processed foods) [33] and the coefficients of environmental footprints [32].

The evaluation of the applicability of principal component analysis (PCA) was performed using Bartlett’s sphericity test (BST) and the Kaiser–Meyer–Olkin (KMO) statistical test. Then, PCA was performed to identify eating patterns, and the number of dietary patterns to retain was determined considering an eigenvalue greater than one, the screen test (i.e., identifying the breaking point in the scree plot of the components’ eigenvalues), and natural interpretability.

Varimax rotation was used to achieve a simple matrix and uncorrelated dietary patterns, and positive factor loading values of ≥0.25 were used to determine the food groups in each dietary pattern. Lastly, a score for each dietary pattern for each individual, based on their intake of different food groups and weighted by corresponding factor loadings, was calculated. The labeling of dietary patterns identified was based on the literature and our interpretation of the data.

### 2.6. Covariates

In the first phase of the questionnaire, data on sex (male/female), age (continuous), individual income (continuous), marital status (single/married/divorced or widow(er)), and professional status (employed/student/retired or home duties/unemployed) were collected. Practice of physical activity was investigated using a list of 23 leisure activities and the respective time dedicated to each one [34]. Participants were classified as physically active, insufficiently active, or inactive, according to the World Health Organization [35].

### 2.7. Statistical Analysis

To characterize the study population, absolute and relative frequencies of the socioeconomic variables were presented for the total sample and according to dietary patterns and the outcomes investigated in this study. For categorical variables, the Pearson chi-square test was used to identify statistical differences between participants’ characteristics. For continuous variables, statistical differences were assessed using the Kruskal–Wallis test and the pairwise comparison of means test with Tukey’s adjustment method.

Linear regression models were performed to evaluate the association between dietary patterns and the components of the global syndemic investigated in this study. Models included factor loadings of the four dietary patterns (Model 1) and were then adjusted for sex, age, individual income, and the daily energy intake in kcal (Model 2). Analysis for the obesity outcome was additionally adjusted for physical activity. All analyses were performed using Stata/SE^®^ 17.0 and adopted a significance level of 5%.

## 3. Results

### 3.1. Characterization of the Sample

Table 1 shows the descriptive statistics of participants according to the global syndemic components investigated in this study. The sample in this study consisted of 7560 participants from the CUME Study, with 68% being female and 64.21% being white. The average age of participants was 35 ± 8.5 years, and their average monthly income was BRL 6108.58 ± 436.49 (approximately USD 1227).

Women were more likely to obesity and typically had inadequate micronutrient intake, while men’s diets emitted a higher amount of CO2e, consumed more water, and required more productive area. Those between the ages of 30 and 49 were more likely to have obesity, insufficient micronutrient intake, and a greater environmental impact. Lower income was associated with obesity and insufficient vitamin intake, whereas environmental footprints increased proportionally with income. Compared to white people’s diets, black/brown people’s diets showed a higher CFP, WFP, and EFP. Regarding marital status, married people’s diets were more likely to cause obesity and have a greater environmental impact. Employed participants were at a higher risk of obesity and insufficient intake of vitamins. Compared to students, employed people’s diets emitted more CO2e and required more productive area. The region of Brazil where the participants lived did not significantly affect the variables investigated here (Table 1).

### 3.2. Dietary Patterns

Regarding the dietary patterns, BTS and KMO tests (BTS ≤ 0.001 and KMO = 0.728) indicated that the sample was suitable for PCA. We identified four dietary patterns (DP), explaining 29% of the data variance. The first pattern, named the Unhealthy DP, consisted of meats, ultra-processed foods, sweetened beverages, and alcoholic beverages. The Brazilian DP comprised rice, beans, culinary ingredients, ultra-processed fats, and white bread. The Healthy DP included eggs, grains and tubers, fruits and natural juice, vegetables, teas and water, nuts, and olive oil. The Dairy DP was composed of milk, cheeses, yogurts, and ultra-processed breads (Table 2).

The Unhealthy DP factor score was higher among men and those with higher incomes. The Brazilian DP factor score was higher among men, older people, those with lower incomes, and unemployed individuals. The Healthy DP factor score increased significantly with age and individual income. The Dairy DP factor score was higher among women and older people. When differences in mean factor scores for dietary patterns were evaluated across Brazil’s regions, we found that the Southeast region had a higher mean factor score for the Brazilian DP and Dairy DP (Table 3).

Overall, the average caloric consumption was 2395.72 ± 11.28 kcal/day and was higher in the Unhealthy DP (2800.82 ± 27.96 kcal/day) and lower in the Dairy DP (2069 ± 18.53). The Unhealthy DP had the highest mean for protein (142.48 ± 1.85 g/day) and total fat (126.25 ± 1.47 g/day), and the Dairy DP had the lowest mean for carbohydrate (232.78 ± 2.23 g/day), protein (92.87 ± 0.90 g/day), and total fat (83.29 ± 0.89 g/day). The fiber content was higher for the Healthy DP (33.69 ± 0.39 g/day).

### 3.3. Malnutrition

**Figure 1:**
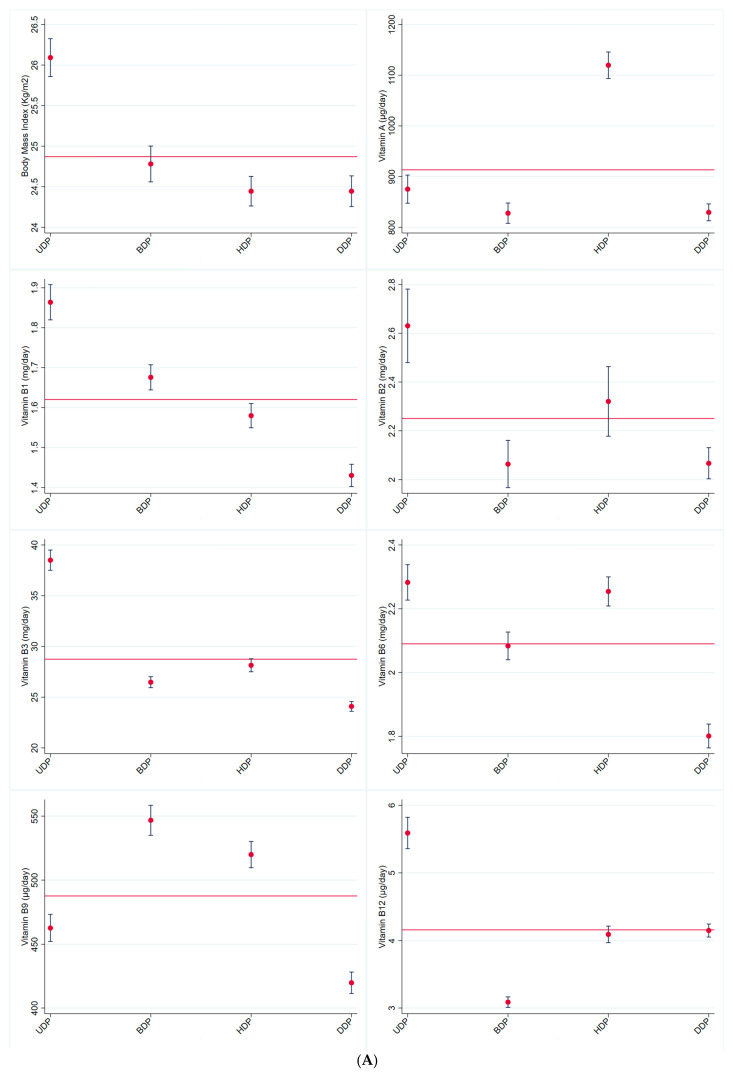

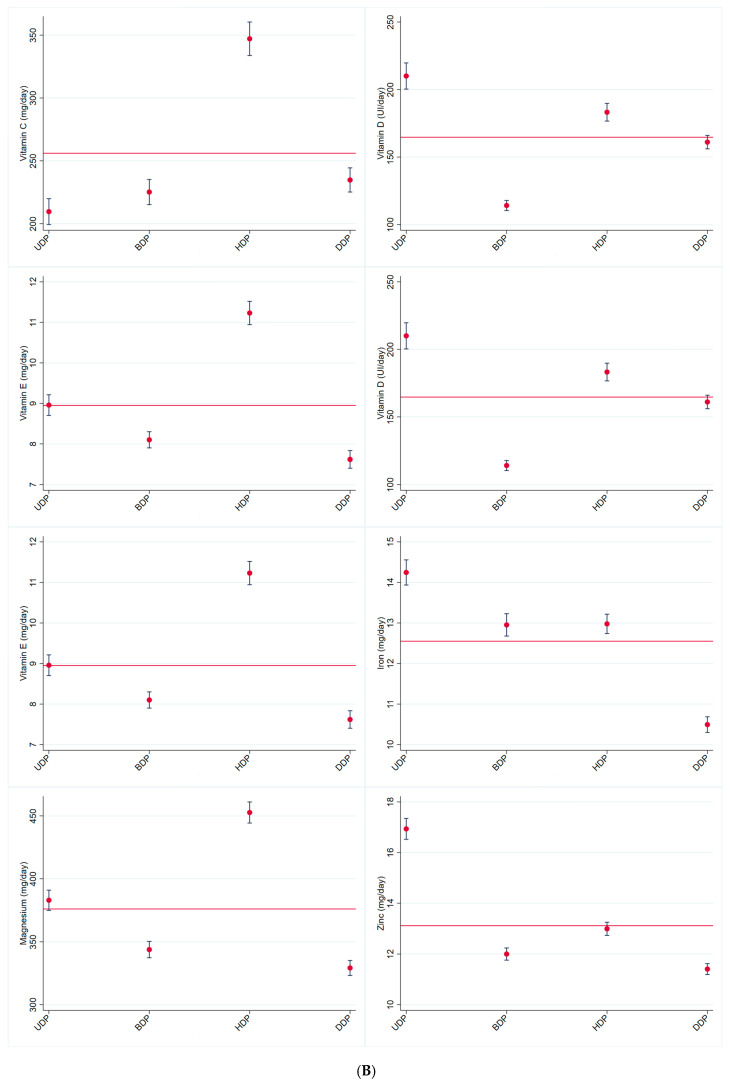

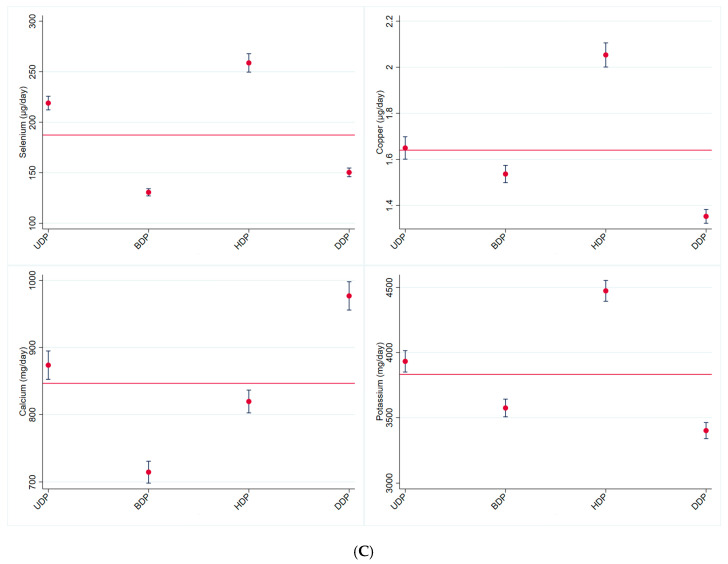
(**A**) Average value (CI 95%) for body mass index (Kg/m^2^) and micronutrient intake (mg or µg/person/day) for the total sample (red line) and according to dietary patterns. (**B**) Average value (CI 95%) for micronutrient intake (mg or µg/person/day) for the total sample (red line) and according to dietary patterns. (**C**) Average value (CI 95%) for micronutrient intake (mg or µg/person/day) for the total sample (red line) and according to dietary patterns. (UDP: unhealthy dietary pattern; BDP: Brazilian dietary pattern; HDP: healthy dietary pattern; DDP: dairy dietary pattern). CUME Study, 2016–2022.

Figure 1 shows the average BMI and micronutrient intake value for the total sample and according to dietary patterns. The mean BMI among those belonging to the Unhealthy DP (26.09 ± 0.12 kg/m^2^) was significantly higher than that for the total sample (24.87 ± 0.05 kg/m^2^).

For the total sample, the daily average intake of vitamins and minerals was adequate, except for vitamins D (164.66 ± 1.64 UI/day) and E (8.95 ± 0.06 mg/day) and Ca (846.51 ± 4.96 mg/day). The Unhealthy DP had the highest daily averages for vitamins B1 (1.86 ± 0.02 mg), B2 (2.63 ± 0.08 mg), B3 (38.50 ± 0.51 mg), B6 (2.28 ± 0.03 mg), B12 (5.59 ± 0.12 µg), and D (210 ± 4.94 UI), as well as Fe (14.25 ± 0.16 mg) and Zn (16.94 ± 0.21 mg). The Brazilian DP had the highest daily average for vitamin B9 (546.75 ± 5.98 µg). The Healthy DP had the highest daily averages for vitamins A (1119.62 ± 13.35 µg), C (347.10 ± 6.80 mg), and E (11.23 ± 0.15 mg), as well as Mg (452.70 ± 4.28 mg), Se (258.65 ± 4.64 µg), Cu (2.05 ± 0.03 µg), and K (4473.90 ± 40.90 mg). The highest daily average for Ca was observed for Dairy DP (976.90 ± 10.77 mg) (Figure 1).

The overall prevalence of insufficient intake of vitamins was 12.49% for vitamin A, 13.08% for vitamin B1, 5.65% for vitamin B2, 3.82% for vitamin B3, 11.40% for vitamin B6, 22.87% for vitamin B9, 16.10% for vitamin B12, 9.01% for vitamin C, 94.51% for vitamin D, and 80.89% for vitamin E. Regarding insufficient intake of minerals, the prevalence was 16.59%, 32.91%, 15.44%, 2.34%, 6.75%, 54.70%, and 29.71% for Fe, Mg, Zn, Se, Cu, Ca, and K, respectively.

The linear regression models displayed in Table 4 were performed to analyze the association between dietary patterns and BMI as well as insufficient intake of micronutrients. After conducting an adjusted analysis, it was found that the Unhealthy DP was associated with a mean increase of 0.95 Kg/m^2^ in BMI. The Brazilian DP was also positively associated with BMI, with a mean increase of 0.24 Kg/m^2^. The Dairy DP showed a discreet positive association with BMI, increasing it by 0.09 Kg/m^2^. The Healthy DP, on the other hand, was inversely associated with BMI, resulting in a mean reduction of 0.12 Kg/m^2^.

Regarding micronutrient intake, it was noted that all four dietary patterns were linked to a lower total number of micronutrient inadequacies, whether assessed as the score for vitamins, minerals, or overall micronutrients. For the total score, the Unhealthy DP decreased by 0.19 micronutrient inadequacies, the Brazilian DP decreased by 0.15 micronutrient inadequacies, the Healthy DP decreased by 0.70 micronutrient inadequacies, and the Dairy DP decreased by 0.57 micronutrient inadequacies (Table 4).

### 3.4. Environmental Footprints

**Figure 2:**
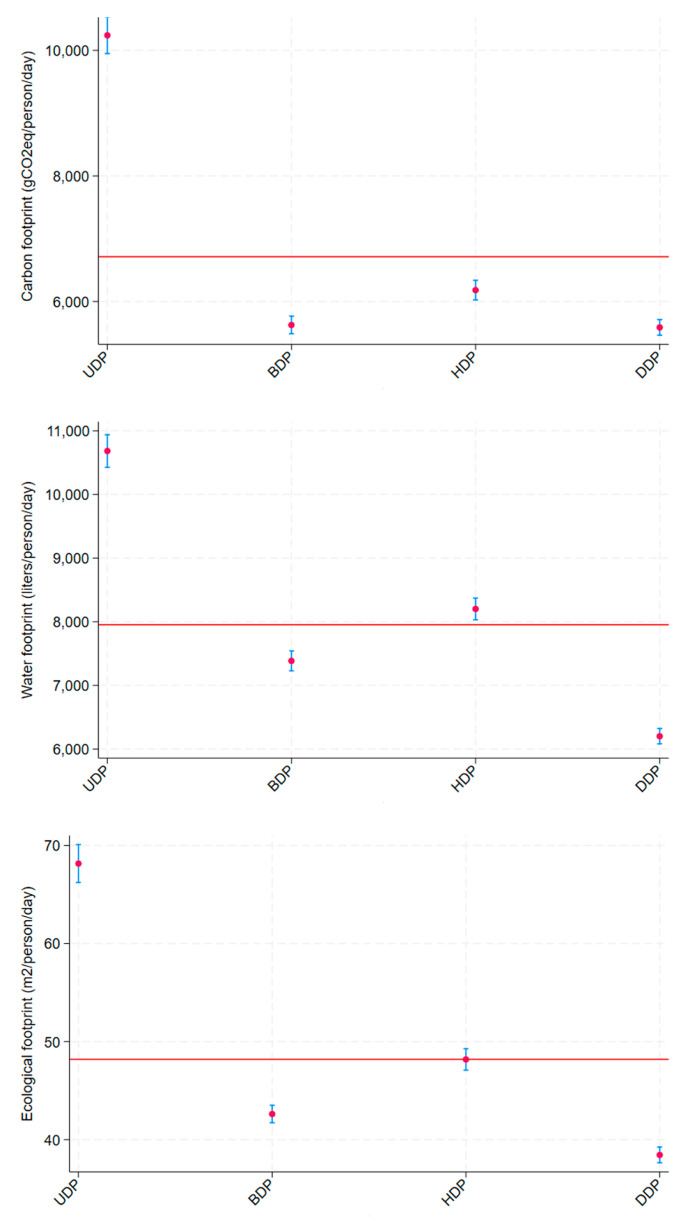
Average value (CI 95%) of the environmental footprints (carbon footprint (gCO2e/person/day), water footprint (L/person/day), and ecological footprint (m^2^/person/day)) according to dietary patterns (UDP: unhealthy dietary pattern; BDP: Brazilian dietary pattern; HDP: healthy dietary pattern; DDP: dairy dietary pattern), with the red line indicating the overall mean of the sample. CUME Study, 2016–2022.

Figure 2 shows the average values of the environmental footprints investigated in this study for the total sample and according to dietary patterns. The red line represents the overall mean of the sample and serves as a reference for comparison. The mean CFP among those belonging to the Unhealthy DP (10,238.44 ± 148.46) was significantly higher than that for the total sample (6713.45 gCOe/person/day). All other dietary patterns showed mean CFP values significantly below the general mean. The mean WFP and EFP for the total sample were 7952.50 L/person/day and 48.19 m^2^/person/day, and they were significantly higher among those belonging to the Unhealthy DP (10,682.86 L/person/day and 68.15 m^2^/person/day, respectively).

The results of the linear regression models found that the Unhealthy DP had the highest environmental impact compared to the other three dietary patterns across all three measurements in both the crude and adjusted analyses. In the adjusted analyses, the CO2e emissions of the Unhealthy DP were 122.3%, 85.7%, and 46.7% higher than those of the Brazilian DP, Healthy DP, and Dairy DP, respectively. Additionally, the Unhealthy DP required 49.7%, 9.2%, and 71% more water than the Brazilian DP, Healthy DP, and Dairy DP, respectively. Regarding the biosphere’s regenerative capacity, the Unhealthy DP required 58.5%, 0.5%, and 30% more bio-productive area than the Brazilian DP, Healthy DP, and Dairy DP, respectively (Table 4).

## 4. Discussion

As far as we know, this study is the first to examine how dietary patterns relate to the three components of the global syndemic. Based on data from the CUME Study, we identified four distinct dietary patterns and labeled them based on the literature and our interpretation of the data. With that in mind, it was expected that the Unhealthy DP and the Dairy DP would be positively associated with all three components of the global syndemic, the Healthy DP would be negatively associated with the three outcomes, and the Brazilian DP would be negatively associated with environmental footprints and present a null or weak association with malnutrition outcomes.

As expected, the Unhealthy DP had the greatest environmental impact for all three metrics used in this study (CFP, WFP, and EFP). This may be explained by its dominant composition of meat and ultra-processed foods. It is well established that animal-source foods require more water and land and emit significantly greater amounts of CO2e [17,36,37]. Recent literature has demonstrated that ultra-processed foods significantly impact CO2e emissions, land use, and water consumption [15,38,39].

The Unhealthy DP also was positively associated with BMI. This dietary pattern consists of food items that are known to contribute to obesity, such as red meats [40,41], ultra-processed foods [42,43], sweetened beverages [40,44], and alcoholic beverages [45,46]. The lower intake of vitamins A, C, and E observed in Figure 1 for the Unhealthy DP was expected, as those nutrients are most commonly found in fresh fruits and vegetables. The higher intake of vitamin B12, Fe, and Zn was expected because of the meat composition of this dietary pattern, as vitamin B12 is exclusively found in animal-source food and meat is a great source of iron and zinc [24,25].

In our study, the Brazilian DP was positively associated with BMI, which was unexpected, as previous studies have shown a null or negative association between traditional Brazilian dietary patterns and obesity measurements [14,47,48]. Studies that investigate dietary patterns on health outcomes are difficult to compare because although dietary patterns may be given the same name, their compositions can vary widely [49]. This is especially true in Brazil, where food culture changes from one region to another, and all of them may be named Brazilian patterns [49,50]. Although not representative of the Brazilian population, CUME Study participants live in all 27 states of Brazil, and the Brazilian DP in this study is composed of rice, beans, culinary ingredients, ultra-processed fats, and white bread. In a study conducted among women in the Brazilian state Rio Grande do Sul, a common Brazilian dietary pattern was characterized by cassava, yam, farofa, chicken, egg, fish, potato, black beans, and polenta [14]. In a study conducted in the state of São Paulo, the traditional Brazilian dietary pattern was composed of positive factorial loading for beans, rice, margarine, and beef and negative factor loading for low-fat dairy foods, whole grain bread, and diet sodas [48]. Both studies showed a negative association between these dietary patterns and excess weight.

Regarding environmental impact, the Brazilian DP had the lowest CFP and EFP and the second lowest WFP of the four dietary patterns studied, showing the low environmental impact of Brazilian dietary patterns on CFP, WFP, and EFP. A previous study estimated that the average CFP of the Brazilian diet in 2008–2009 was 4489 gCO2e/person/day [51], which is 0.33 times lower than the mean CFP for the total sample and 0.20 times lower than the mean CFP for the Brazilian DP in this study. Although participants may have belonged to the Brazilian DP, it does not mean that they do not consume meat and other food items with higher CFP. Our results show that CFP tends to increase with income, which is consistent with the results of Garzillo et al. Moreover, Garzillo et al. found that CFP increases with higher levels of education, which may explain the higher CFP observed in our study, given that all participants were university graduates.

Although the Brazilian DP had the lowest environmental impact in this study, international literature suggests there are diets with even lower environmental impacts. While this Brazilian DP emits 5627.15 gCO2e/person/day, the current Indonesian diet, mainly composed of grains, starchy roots, and some animal-source food, emits less than 3000 gCO2e/person/day [52]. Data from Seguimiento Universidad de Navarra (SUN Cohort) in Spain estimated dietary emissions at 3540 gCO2e/person/day [53]. In our study, the Brazilian DP required 7384.44 L/person/day of water and 42.61 m^2^/person/day of land, whereas the Spanish diet required only 3753 L/person/day of water and 7.17 m^2^/person/day [53]. On the other hand, data from the NHANES Study estimated 2230 gCO2e/1000 kcal [54], a result similar to ours. The discrepancies observed may be due to diet composition and the different data sources used in these studies, which account for the different food production systems.

The Brazilian diet is known for its variety, consisting mainly of unprocessed or minimally processed foods, along with plant-based food combinations complemented by minimal animal-source food. If followed properly, it can provide a balanced and nutritious diet rich in vitamins, minerals, bioactive compounds, and fiber, which can help prevent obesity and micronutrient deficiencies. It also promotes a fairer food system, supporting the local economy and respecting food culture. Additionally, limiting animal-source food consumption reduces the environmental impact on the food system [50]. The absence of animal-source food in the Brazilian DP in our study may mean that its consumption is limited. On the other hand, the lack of fruits and vegetables may mean that the Brazilian diet is not as varied as it should be and does not follow Brazilian dietary guidelines.

Recent studies in Brazil have examined the connection between dietary patterns and obesity across various populations, adding to the expanding national evidence on the subject. Analyses from both longitudinal and cross-sectional cohorts have found that diets high in ultra-processed foods and convenience products are significantly linked to increased markers of adiposity and obesity. Conversely, diets featuring traditional or healthy foods tend to show protective benefits [48,55,56,57]. Nonetheless, these findings should be approached with caution, as the makeup of dietary habits can differ widely between studies, particularly in Brazil, where food culture is highly regionalized, and similar names may refer to distinct eating patterns.

In this study, the Healthy DP was the most protective against inadequacy of micronutrients. This was expected, as it is a diversified dietary pattern composed mainly of fresh fruits and vegetables, a great source of vitamins such as C and E and minerals such as K [24,25]. Additionally, Figure 1 shows that the Dairy DP had the highest intake of Ca, which is not surprising, as dairy products are the primary source of this mineral [24,25]. It was not surprising that the Healthy DP had the second lowest CFP, as research has shown that plant-based foods generally have a lower CFP [36,38,58,59]. However, some results for the environmental impacts of the Healthy DP and Dairy DP were thought-provoking. The Healthy DP had the second-highest WFP, while the Dairy DP had the lowest WFP. In agreement with our study, Berardy et al. [38] estimated the environmental impacts of food groups and reported a lower water consumption for dairy products and high water consumption for fruits, a major food item in our Healthy DP. They also reported that nuts and seeds, food items also belonging to our Healthy DP, had the highest water consumption on a protein content basis, probably due to the small edible mass compared to the overall plant biomass.

Besides that, WFP is often reported as a single value in the literature, but it is composed of three components—green water, blue water, and grey water—green water being the amount of precipitation and soil moisture that is directly consumed in growing crops [32,60]. Around three-quarters of the global water footprint is green water [60], which may explain the greater WFP for the Healthy DP. Rainfed agriculture accounts for over 80% of cropland worldwide and produces more than 60% of all cereal grains. Therefore, using green water may not significantly impact the overall water budget [61,62].

Moreover, food items that comprise the Healthy DP are less calorically dense [24,25], which could lead to increased overall food consumption, since environmental impacts were analyzed here for each gram of edible food consumed by CUME Study participants. On average, participants belonging to the Healthy DP consumed the greatest amount of food (3653.1 ± 22.71 g of food/person/day), while those belonging to the Dairy DP had the lowest total food intake (2805.44 ± 20.77 g of food/person/day).

It is worth mentioning that there is currently no food production with zero or negative CFP, WFP, or EFP. Therefore, the negative coefficient values for environmental footprint in Table 4 mean that, for the four dietary patterns being compared, the negative values represent the difference between them. Further research is needed to identify diverse compositions of a traditional Brazilian dietary pattern and their impact on the components of the global syndemic.

A strength of our study is that the CUME Study uses an online platform, which enabled us to recruit participants from all regions of Brazil. Our sample consisted of individuals with a high level of education, which ensured the reliability of the data, as it was self-reported. Also, food consumption data were obtained using a validated FFQ [19], as well as data regarding BMI [22]. Although this study included a highly educated sample, which does not represent the Brazilian population, representativeness is usually unnecessary in analytical epidemiological studies [63]. Thus, the CUME Study provides data that allow the discussion, planning, and implementation of specific interventions in Brazil. Another strength is that we assessed three metrics of the environmental impact of dietary patterns consumed by Brazil’s population. We also used environmental impact coefficients that consider the entire life cycle of foods, from “farm to fork”.

A limitation of this study is that although the environmental impact coefficients used in the study were calculated based on food and culinary preparations consumed in Brazil, some of their estimates came from studies conducted in other countries and industry reports. Also, our sample population allowed us to evaluate the outcome of undernutrition only in terms of insufficient intake of micronutrients, without considering underweight and food safety factors.

## 5. Conclusions

For the first time, the relationship between dietary patterns and the three components of the global syndemic was explored. In this Brazilian university graduate sample, diet affected the environment and people’s health. Obesity and micronutrient inadequacy prevalence was high, while CFP, WFP, and EFP were higher than the national average and that of other countries.

Our study highlights the need to understand the different compositions of the Brazilian dietary pattern and how they may affect the three components of the global syndemic. Our results also reveal that the Brazilian diet is not in line with the Brazilian Dietary Guidelines, which negatively affects the environment and people’s health. Therefore, it is important for health services to educate the general population about the importance of following the guidelines for better health and a healthier environment. At the same time, the dietary patterns identified here as contributing to poor health and environmental damage can help the government develop policies that incorporate the costs of these effects into the prices of the food.

## Figures and Tables

**Table 1:** Sociodemographic characteristics, according to global syndemic components, baseline CUME Study (*n* = 7560, 2016–2022).

Characteristic	Total *n* (%)	Obesity *n* (%)	VII *n* (%)	MII *n* (%)	CFP Mean ± sd	WFP Mean ± sd	EFP Mean ± sd
Sex							
Male	2420 (32.01)	380 (40.25)	2328 (31.71)	1454 (30.85)	7676.13 ± 94.5 ^a^	9106 ± 93.12 ^a^	54.72 ± 0.62 ^a^
Female	5140 (67.99)	564 (59.75)	5014 (68.29)	3259 (69.15)	6260.20 ± 56.53 ^b^	7409.41 ± 53.15 ^b^	45.11 ± 0.37 ^b^
*p*-value	-	**<0.001**	**0.001**	**0.005**	-	-	-
Age							
20–29	2317 (30.65)	175 (18.54)	2266 (30.86)	1454 (30.85)	6364.07 ± 88.76 ^a^	7499.17 ± 85.6 ^a^	44.53 ± 0.54 ^a^
30–39	3234 (42.78)	382 (40.47)	3156 (42.99)	2046 (43.41)	6811.93 ± 76.36 ^b^	8020.24 ± 72.7 ^b^	48.9 ± 0.52 ^b^
40–49	1388 (18.36)	261 (27.65)	1338 (18.22)	819 (17.38)	7030.4 ± 113.9 ^b^	406.16 ± 111.37 ^c^	51 ± 0.72 ^b^
50–59	621 (8.21)	126 (13.35)	582 (7.93)	394 (8.36)	6795.77 ± 171.57 ^a,b^	8277.12 ± 167.7 ^b,c^	51.86 ± 1.17 ^b^
*p*-value	-	**<0.001**	**<0.001**	**0.041**	-	-	-
Individual income (minimum wage) *							
<5	4344 (57.46)	499 (52.86)	4225 (57.55)	2742 (58.18)	6430.43 ± 65.97 ^a^	7704.42 ± 63.5 ^a^	45.92 ± 0.42 ^a^
≥5 to <10	2215 (29.30)	273 (28.92)	2160 (29.42)	1365 (28.96)	7002.66 ± 91.38 ^b^	8194.37 ± 86.49 ^b^	50.3 ± 0.61 ^b^
≥10	1001 (13.24)	172 (18.22)	957 (13.03)	606 (12.86)	7301.7 ± 126.73 ^b^	8493.87 ± 129.1 ^b^	53.34 ± 0.83 ^c^
*p*-value	-	**<0.001**	**0.008**	0.224	-	-	-
Skin color							
White	4854 (64.21)	579 (61.33)	4722 (64.31)	2993 (63.51)	6532.94 ± 56.85 ^a^	7813.05 ± 55.95 ^a^	47.36 ± 0.38 ^a^
Black/Brown	2614 (34.58)	349 (36.97)	2530 (34.46)	1659 (35.20)	7024.65 ± 94.35 ^b^	8192.58 ± 88.46 ^b^	49.55 ± 0.6 ^b^
Yellow/Indigenous	925 (1.22)	16 (1.69)	90 (1.23)	61 (1.29)	7395.27 ± 560.77 ^a,b^	8488.7 ± 564.64 ^a,b^	53.36 ± 3.74 ^a,b^
*p*-value	-	0.074	0.439	0.225	-	-	-
Marital status							
Single	3702 (84.97)	390 (41.31)	3604 (49.09)	2311 (49.03)	6569.68 ± 71.79 ^a^	7798.04 ± 69.27 ^a^	46.75 ± 0.47 ^a^
Married	3500 (46.30)	501 (53.07)	3392 (46.20)	2169 (46.02)	6888.26 ± 71.48 ^b^	8099.86 ± 68.69 ^b^	49.63 ± 0.47 ^b^
Divorced/Widow(er)	358 (4.74)	53 (5.61)	346 (4.71)	233 (4.94)	6491.12 ± 227.53 ^a,b^	8109.01 ± 224.62 ^a,b^	48.9 ± 1.4 ^a,b^
*p*-value	-	**<0.001**	0.465	0.506	-	-	-
Professional status							
Employed	5611 (74.22)	748 (79.24)	5447 (74.19)	3538 (75.07)	6789.75 ± 56.95 ^a^	8016.13 ± 54.83 ^a^	48.74 ± 0.37 ^a^
Student	1292 (17.09)	103 (10.91)	1265 (17.23)	779 (16.53)	6415.72 ± 122.02 ^b^	7754.35 ± 118.65 ^a^	46.29 ± 0.84 ^b^
Retired/home duties	96 (1.27)	21 (2.22)	94 (1.28)	65 (1.38)	6913.39 ± 494.5 ^a,b^	7914.73 ± 411.94 ^a^	48.29 ± 2.5 ^a,b^
Unemployed	591 (7.42)	72 (7.63)	536 (7.30)	331 (7.02)	6601.82 ± 184.46 ^a,b^	7778.87 ± 181.81 ^a^	47.06 ± 1.26 ^a,b^
*p*-value	-	**<0.001**	**0.044**	0.061	-	-	-
Region of Brazil							
North	120 (1.38)	13 (1.38)	102 (1.39)	69 (1.47)	7077.48 ± 467.56 ^a^	7712.54 ± 424.09 ^a^	50.55 ± 2.75 ^a^
Northeast	274 (3.16)	33 (3.50)	229 (3.13)	151 (3.21)	7152.16 ± 288.10 ^a^	8019.95 ± 258.71 ^a^	52.22 ± 1.87 ^a^
Southeast	7824 (90.18)	839 (89.07)	6605 (90.16)	4208 (89.53)	6680.99 ± 52.17 ^a^	7952 ± 50.48 ^a^	48.01 ± 0.34 ^a^
Midwest	304 (3.50)	39 (4.14)	258 (3.52)	183 (3.89)	7141.98 ± 275.26 ^a^	8193.49 ± 268.56 ^a^	49.95 ± 1.62 ^a^
South	154 (1.78)	18 (1.91)	132 (1.80)	89 (1.89)	6473.98 ± 281.42 ^a^	7649.92 ± 265.73 ^a^	45.49 ± 1.66 ^a^
*p*-value	-	0.798	0.253	0.186	-	-	-

*p*-Values were obtained from the Pearson chi-squared test for categorical variables, and bold indicates statistical significance (*p* ≤ 0.05). Differences for continuous variables were obtained using the Kruskal–Wallis test or pairwise comparison of means with Tukey’s adjustment method; different letters indicate statistically significant differences (*p* ≤ 0.05). VII: insufficient intake of at least one vitamin; MII: insufficient intake of at least one mineral; CFP: carbon footprint; WFP: water footprint; EFP: ecological footprint. * Minimum wage (R$ 880.00 in 2016; R$ 954.00 in 2018; R$ 1045.00 in 2020; R$ 1212.00 in 2022).

**Table 2:** Dietary patterns and factor loadings of food groups consumed by CUME Study participants at baseline (*n* = 7560, 2016–2022).

Food Groups	Dietary Patterns				*h* ^2^
	Unhealthy	Brazilian	Healthy	Dairy	
Milk	-	-	-	0.4524	0.6854
Cheeses	-	-	-	0.2820	0.7623
Yogurts	-	-	-	0.4280	0.6468
Red meat	0.3990	-	-	-	0.6726
Chicken and fish	0.3223	-	-	-	0.6509
Salt meat	0.2966	-	-	-	0.8127
Sushi	-	-	-	-	0.8869
Egg	-	-	0.3264	-	0.7011
Ultra-processed meats	0.3995	-	-	-	0.6411
Rice and pasta	-	0.4337	-	-	0.5997
Beans	-	0.3084	-	-	0.6701
Grains and tubers	-	-	0.3047	-	0.6277
Fried tubers	-	-	-	-	0.8036
Culinary ingredients	-	0.3655	-	-	0.716
Ultra-processed fats	-	0.2618	-	-	0.7317
Fruits and natural juice	-	-	0.4080	-	0.5694
Vegetables	-	-	0.4553	-	0.5518
Coffee	-	-	-	-	0.8352
Teas and water	-	-	0.3161	-	0.7882
Sweetened beverages	0.2899	-	-	-	0.7121
Fermented alcoholic beverages	0.2743	-	-	-	0.6865
Distilled alcoholic beverages	0.2608	-	-	-	0.7732
Nuts and olive oils	-	-	0.3403	-	0.7532
*French* bread	-	0.3782	-	-	0.6359
Ultra-processed breads	-	-	-	0.3555	0.7101
Preserves (jams)	-	-	-	-	0.9175
Ultra-processed foods	0.3059	-	-	-	0.6775

**Table 3:** Sociodemographic characteristics, according to dietary patterns, baseline CUME Study (*n* = 7560, 2016–2022).

Characteristic	Total *n* (%)	Dietary Patterns			
		Unhealthy (UDP)	Brazilian (BDP)	Healthy (HDP)	Dairy (DDP)
Sex					
Male	2420 (32.01)	0.46 ± 0.03 ^a^	0.39 ± 0.03 ^a^	0.05 ± 0.03 ^a^	−0.14 ± 0.03 ^a^
Female	5140 (67.99)	−0.22 ± 0.02 ^b^	−0.19 ± 0.02 ^b^	−0.02 ± 0.02 ^a^	0.07 ± 0.02 ^b^
Age					
20–29	2317 (30.65)	−0.02 ± 0.03 ^a^	−0.02 ± 0.03 ^a^	−0.15 ± 0.03 ^a^	0.00 ± 0.02 ^a,b^
30–39	3234 (42.78)	0.03 ± 0.03 ^a^	−0.06 ± 0.02 ^a^	−0.03 ± 0.02 ^b^	−0.06 ± 0.02 ^b^
40–49	1388 (18.36)	0.02 ± 0.04 ^a^	0.15 ± 0.04 ^b^	0.13 ± 0.04 ^c^	0.07 ± 0.03 ^a,c^
50–59	621 (8.21)	−0.13 ± 0.06 ^a^	0.02 ± 0.06 ^a,b^	0.39 ± 0.06 ^d^	0.15 ± 0.05 ^c^
Individual income *					
<5	4344 (57.46)	−0.06 ± 0.02 ^a^	0.08 ± 0.02 ^a^	−0.05 ± 0.02 ^a^	0.00 ± 0.02 ^a^
≥5 to <10	2215 (29.30)	0.06 ± 0.03 ^b^	−0.09 ± 0.03 ^b^	0.04 ± 0.03 ^b^	0.02 ± 0.03 ^a^
≥10	1001 (13.24)	0.16 ± 0.05 ^b^	−0.14 ± 0.04 ^b^	0.13 ± 0.04 ^b^	−0.04 ± 0.04 ^a^
Skin color					
White	4854 (64.21)	−0.04 ± 0.02 ^a^	−0.07 ± 0.02 ^a^	0.00 ± 0.02 ^a^	0.00 ± 0.02 ^a^
Black/Brown	2614 (34.58)	0.06 ± 0.03 ^b^	0.13 ± 0.03 ^b^	−0.00 ± 0.03 ^a^	0.00 ± 0.02 ^a^
Yellow/Indigenous	925 (1.22)	0.13 ± 0.18 ^a,b^	0.14 ± 0.16 ^a,b^	0.04 ± 0.13 ^a^	−0.14 ± 0.11 ^a^
Marital status					
Single	3702 (84.97)	0.03 ± 0.02 ^a^	−0.01 ± 0.02 ^a^	−0.05 ± 0.02 ^a^	0.01 ± 0.02 ^a^
Married	3500 (46.30)	−0.02 ± 0.02 ^a^	0.01 ± 0.02 ^a^	0.03 ± 0.02 ^b^	−0.01 ± 0.02 ^a^
Divorced/Widow (e)	358 (4.74)	−0.09 ± 0.08 ^a^	0.00 ± 0.07 ^a^	0.17 ± 0.08 ^b^	0.07 ± 0.07 ^a^
Professional status					
Employed	5611 (74.22)	0.02 ± 0.02 ^a^	−0.03 ± 0.02 ^a^	0.01 ± 0.02 ^a,b,c^	−0.02 ± 0.02 ^a^
Student	1292 (17.09)	−0.03 ± 0.04 ^a^	0.04 ± 0.04 ^a^	−0.04 ± 0.04 ^b^	0.04 ± 0.03 ^a^
Retired/home duties	96 (1.27)	−0.45 ± 0.12 ^b^	−0.24 ± 0.13 ^a^	0.37 ± 0.14 ^c^	0.15 ± 0.12 ^a^
Unemployed	591 (7.42)	−0.02 ± 0.07 ^a^	0.25 ± 0.07 ^b^	−0.02 ± 0.06 ^a,b,c^	0.08 ± 0.05 ^a^
Region of Brazil					
North	120 (1.38)	−0.03 ± 0.17 ^a^	−0.23 ± 0.13 ^a,b,c^	−0.06 ± 0.13 ^a^	−0.11 ± 0.11 ^a,b,c^
North-east	274 (3.16)	0.08 ± 0.10 ^a^	−0.14 ± 0.09 ^a,b,c^	0.25 ± 0.10 ^a^	−0.03 ± 0.08 ^b^
South-east	7824 (90.18)	−0.01 ± 0.02 ^a^	0.03 ± 0.02 ^b^	−0.01 ± 0.02 ^a^	0.02 ± 0.01 ^a,b^
Mid-west	304 (3.50)	0.10 ± 0.09 ^a^	−0.29 ± 0.08 ^c^	0.10 ± 0.09 ^a^	−0.33 ± 0.07 ^c^
South	154 (1.78)	−0.03 ± 0.11 ^a^	−0.24 ± 0.11 ^a,b,c^	−0.26 ± 0.12 ^a^	−0.08 ± 0.11 ^a,b,c^

Differences were obtained using the Kruskal–Wallis test or pairwise comparison of means with Tukey’s adjustment method, and different letters indicate statistically significant differences (*p* ≤ 0.05). * Minimum wage (R$ 880.00 in 2016; R$ 954.00 in 2018; R$ 1045.00 in 2020; R$ 1212.00 in 2022).

**Table 4:** Regression models for evaluating the association of dietary patterns with components of the global syndemic (obesity, insufficient intake of micronutrients, and environmental footprints) in baseline CUME Study participants (*n* = 7560, 2016–2022).

Outcome	Dietary Patterns Coefficient * (95% Confidence Interval)							
	Unhealthy (UDP)		Brazilian (BDP)		Healthy (HDP)		Dairy (DDP)	
	Model 1	Model 2	Model 1	Model 2	Model 1	Model 2	Model 1	Model 2
Body Mass Index	0.70 (0.62–0.78)	0.94 (0.80–1.08)	0.13 (0.05–0.21)	0.20 (0.10–0.31)	−0.06 (−0.12–0.01)	0.16 (0.05–0.27)	−0.05 (−0.14–0.03)	0.14 (0.04–0.24)
Insufficient intake								
Vitamin score	−0.34 (−0.36–−0.32)	−0.11 (−0.15–−0.07)	−0.24 (−0.27–−0.22)	−0.10 (−0.13–−0.06)	−0.58 (−0.61–−0.55)	−0.37 (−0.41–−0.33)	−0.40 (−0.43–−0.37)	−0.27 (−0.30–−0.24)
Mineral score	−0.34 (−0.36–−0.32)	−0.09 (−0.13–−0.05)	−0.21 (−0.23–−0.18)	−0.05 (−0.08–−0.02)	−0.55 (−0.58–−0.53)	−0.33 (−0.37–−0.29)	−0.45 (−0.48–−0.42)	−0.30 (−0.33–−0.27)
Total score	−0.68 (−0.72–−0.64)	−0.19 (−0.26–−0.12)	−0.45 (−0.49–−0.41)	−0.15 (−0.20–−0.09)	−1.13 (−1.19–−1.09)	−0.70 (−0.77–−0.63)	−0.85 (−0.90–−0.80)	−0.57 (−0.62–−0.51)
Environmental data								
CFP	2145.82 (2062.65–2228.99)	803.42 (680.09–926.74)	−121.33 (−179.30–−63.36)	−983.02 (−1086.64–−879.40)	432.92 (383.53–482.30)	−689.21 (−804.99–−573.42)	283.73 (214.66–352.80)	−375.37 (−463.86–−286.88)
WFP	1879.49 (1809.02–1949.96)	843.32 (741.13–945.50)	248.90 (192.54–305.26)	−419.22 (−504.32–−334.11)	941.87 (889.68–994.06)	78.02 (−13.29–169.34)	−99.19 (−168.65–−29.74)	−599.08 (−676.77–−521.38)
EFP	13.36 (12.77–13.95)	7.33 (6.52–8.14)	−0.37 (−0.79–0.05)	−4.29 (−4.98–−3.61)	5.19 (4.85–5.54)	0.04 (−0.61–0.69)	0.79 (0.34–1.25)	−2.21 (−2.73–−1.68)

Model 1: Multiple regression models, considering all four dietary patterns; Model 2: Model 1 adjusted for sex, age, income, and total energy (kcal); analysis for body mass index was additionally adjusted for physical activity. CFP: carbon footprint (gCO2eq/person/day); WFP: water footprint (liters/person/day); EFP: ecological footprint (m^2^/person/day). * Odds Ratio (OR) for logistic regression.

## Data Availability

The raw data supporting the conclusions of this article will be made available by the authors upon request.
